# Differences in long-term survival outcomes after coronary artery bypass grafting using single vs multiple arterial grafts: a meta-analysis with reconstructed time-to-event data and subgroup analyses

**DOI:** 10.1007/s11748-022-01891-7

**Published:** 2022-11-17

**Authors:** Dimitrios E. Magouliotis, Maria P. Fergadi, Prokopis-Andreas Zotos, Arian Arjomandi Rad, Andrew Xanthopoulos, Metaxia Bareka, Kyriakos Spiliopoulos, Thanos Athanasiou

**Affiliations:** 1 Department of Cardiothoracic Surgery, University of Thessaly, Biopolis, 41110 Larissa, Greece; 2 Department of Radiology, University of Thessaly, Larissa, Greece; 3 Department of Surgery and Cancer, Imperial College London, London, UK; 4 Department of Cardiology, University of Thessaly, Larissa, Greece; 5 Department of Anesthesiology, University of Thessaly, Larissa, Greece

**Keywords:** Cabg, Multiple arterial grafting, Mag, Sag, Meta-analysis

## Abstract

**Objective:**

We reviewed the available literature on patients with coronary artery disease undergoing isolated coronary artery bypass grafting (CABG) with either single (SAG) or multiple arterial grafting (MAG).

**Methods:**

Original research studies that evaluated the long-term survival of MAG versus SAG were identified, from 1995 to 2022. The median overall survival (OS) and event-free OS were the primary endpoints. Comparison of median OS between the right internal mammary artery (RIMA) and radial artery (RA) as a second arterial conduit was the secondary endpoint. Subgroup analyses were performed regarding patients older than 70 years, with diabetes mellitus, and females. A sensitivity analysis was performed with the leave-one-out method.

**Results:**

Forty-four studies were included in the qualitative and thirty-nine in the quantitative synthesis. After pooling data from 180 to 459 patients, the MAG group demonstrated a higher OS (HR, 0.589; 95% CI, 0.58–0.60; *p* < 0.0001) and event-free OS compared with the SAG group (HR, 0.828; 95% CI, 0.80–0.86; *p* < 0.0001). In addition, RITA was associated with superior OS compared with RA as a second arterial conduit (HR, 0.936; 95% CI, 0.89–0.98; *p* = 0.009). MAG was also superior to SAG in patients over 70 years, females, and patients with diabetes mellitus. Sensitivity analysis demonstrated a small-size study effect on the female subgroup analysis.

**Conclusion:**

The present meta-analysis indicates that MAG is associated with enhanced survival outcomes compared to SAG for patients undergoing isolated CABG.

**Supplementary Information:**

The online version contains supplementary material available at 10.1007/s11748-022-01891-7.

## Introduction

Despite the progress in cardiac surgery, whether coronary artery bypass grafting (CABG) should be performed with multiple arterial grafts remains highly debated. Numerous observational studies and meta-analyses have reported the benefit of using multiple arterial grafting (MAG) [1–3]. The radial artery (RA), the right internal thoracic artery (RITA), along with the saphenous vein (SV) are all grafts that are routinely being used, although a significant part of the surgeons still favors the use of SV. The main reason is that previous RCTs have failed to demonstrate a survival benefit of MAG over single arterial grafting (SAG) because they were either underpowered [4] or inconclusive due to discrepancies between the treatment allocated and the treatment that was received [5]. Nonetheless, a recently published post hoc analysis of the SYNTAXES trial has demonstrated the superiority of MAG over SAG for patients undergoing CABG [6]. In the same context, the results of the ongoing ROMA trial comparing MAG with single internal thoracic artery (SITA) grafting, which was conceptualized to address the drawbacks of ART mentioned above, are not expected until 2025 [7].

Although there is a previous meta-analysis on the topic [3], it failed to provide any sensitivity analysis, subgroup analyses regarding diabetes and sex were not performed, it did not use independent patient data and no Kaplan–Meier curves were constructed. To provide credible evidence on this topic in the interim period until the publication of ROMA outcomes, we decided to perform a meta-analysis on long-term survival endpoints comparing MAG and SAG as two different CABG strategies for patients with coronary artery disease (CAD), using independent patient data, thus enhancing the level of evidence.

## Materials and methods

### Search strategy and articles selection

The present study was conducted according to the protocol agreed by all authors and the Preferred Reporting Items for Systematic Reviews and Meta-Analyses [8]. A thorough literature search in Pubmed (Medline), Scopus (ELSEVIER), and Cochrane Central Register of Controlled Studies (CENTRAL) (last search: October 18th, 2022) was performed. The following terms were employed in every possible combination: “coronary artery bypass grafting”, “cabg”, “multiple arterial grafting”, “multiple arterial graft”, “multiple arteries”, “mag”, “single arterial graft”, “sag”, “radial artery”, “ra”, “right internal thoracic artery”, “rita”, “rima”, “sima”, “bima”, and “bilateral internal mammary artery”. Inclusion criteria were (1) original reports with ≥ 10 patients, (2) written in English, (3) published from 1995 to 2022, (4) conducted on human subjects, and (5) reporting outcomes of patients with CAD undergoing isolated CABG with either MAG or SAG (SAG was defined as the anastomosis of left internal thoracic artery (LITA) to the left anterior descending (LAD) arterial target). Duplicate articles were excluded. The reference lists of all included articles were also reviewed for additional studies. Two independent reviewers (DEM, MPF) extracted data from the included studies. Any discrepancies between the investigators were discussed with the senior author (TA) to include articles that best matched the criteria until consensus was reached. The authors had personal equipoise regarding the best intervention.

### Data extraction and endpoints

For each eligible study, data were extracted relative to demographics (number of patients, gender, age, ejection fraction (EF), comorbidities, the use of either off-pump (OPCAB) or on-pump coronary artery bypass (ONCAB), and follow-up), along with the long-term survival endpoints (median overall survival (OS) and median event-free survival). Although multiple studies analyzed the same population, only the larger study or the one with the longest follow-up was included.

Median OS and event-free OS were the primary endpoints. Event-free OS was defined as OS free of a major adverse cardiac and cerebrovascular event (MACCE) or reintervention/reoperation. Median OS in patients receiving either the right internal mammary artery (RIMA) or radial artery (RA) as a second arterial conduit was the secondary endpoint. Pooled analysis of overall survival was performed based on the published Kaplan–Meier graphs from the included studies, using the 2-stage approach as described by Liu et al. [9]. In the first stage, raw data coordinates (time, survival probability) were extracted from each treatment arm in the Kaplan–Meier curves. In the second stage, the data coordinates were processed based on the raw data coordinates from the first stage in conjunction with the numbers at risk at certain time points, and individual patient data (IPD) were reconstructed. Finally, the reconstructed IPD were pooled and visualized in Kaplan–Meier graphs. The Gehan–Breslow–Wilcoxon test was employed to compare the OS and event-free OS between the two groups. A *p *value < 0.05 was set as the threshold indicating a statistically significant result. Finally, the Mantel–Haenszel method was employed to calculate the hazard ratio (HR) with 95% confidence intervals (95% CI).

### Sensitivity analysis on primary endpoints

To further validate our outcomes, we performed additional sensitivity analyses regarding OS and DFS using the leave-one-out method. The leave-one-out method involves performing a meta-analysis on each subset of the studies obtained by leaving out exactly one study. Furthermore, we constructed Kaplan–Meier curves using adjusted patient groups regarding OS to further assess our outcomes. Finally, we performed subgroup analyses on females, patients aged > 70 years, and patients with diabetes mellitus (DM).

### Quality and publication bias assessment

The Newcastle–Ottawa Quality Assessment Scale (NOS) [10] was used as an assessment tool to evaluate non-RCTs. The scale’s range varies from zero to nine stars, and studies with a score equal to or higher than five were considered to have adequate methodological quality. The Risk of Bias in Non-Randomized Studies of Interventions tool (ROBINS-I) was also systematically used to assess the included studies for risk of bias [11]. The RCTs were assessed for their quality according to the Cochrane Handbook for Systematic Reviews of Interventions [12]. Two reviewers (DEM, MPF) rated the studies independently and a final decision was reached by consensus.

## Results

### Search strategy and patient demographics

**Fig. 1:**
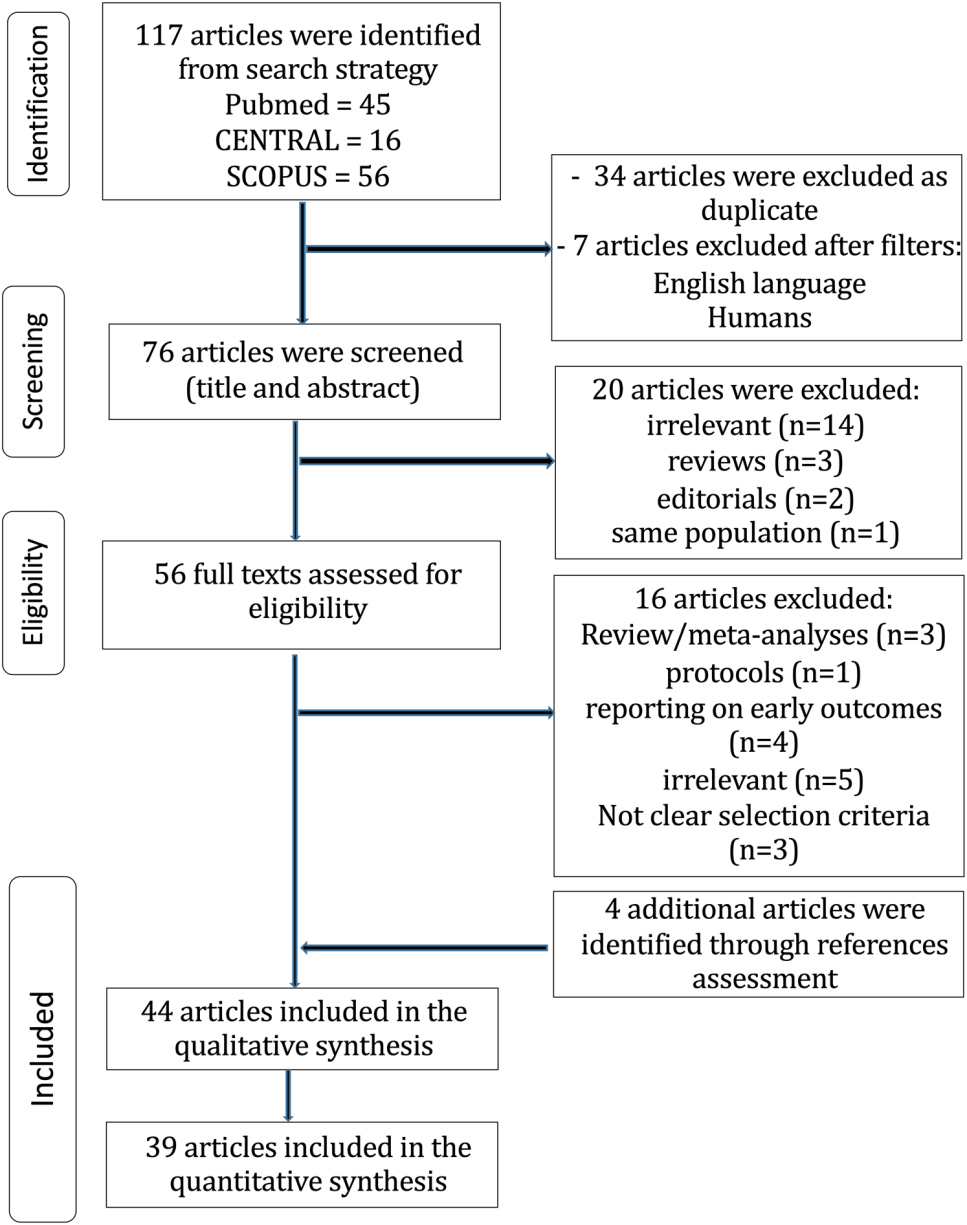
MAG vs. SAG trial flow

**Fig. 2:**
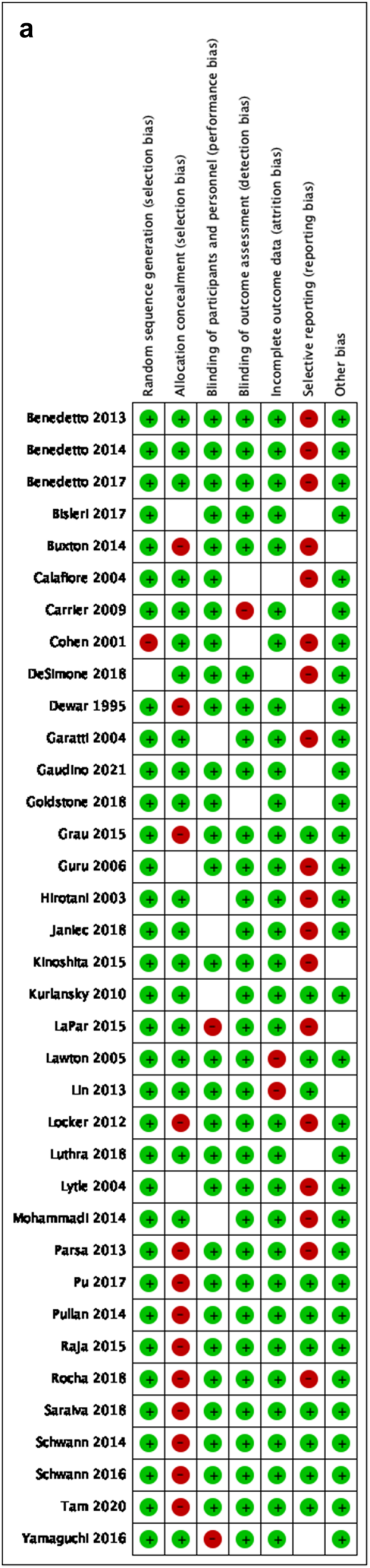

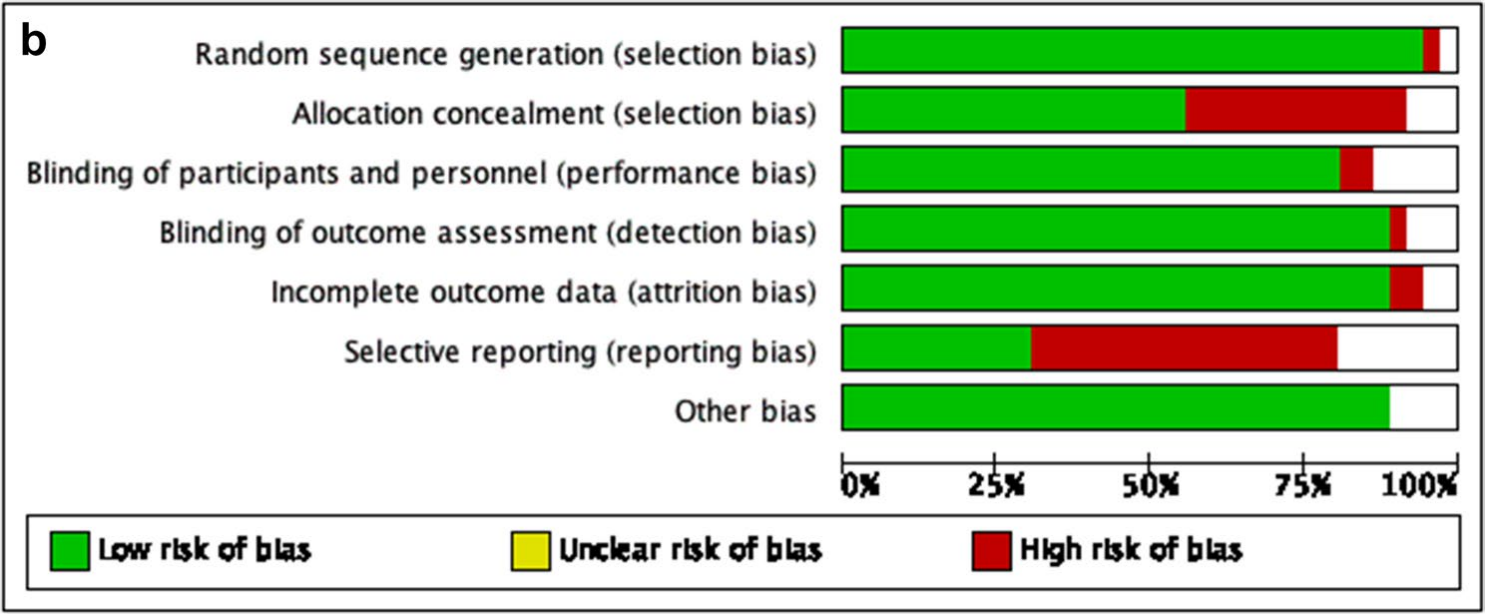
Risk of Bias in Non-Randomized Studies of Interventions with **a**. traffic lights. **b**. summary plot

The flow diagram regarding the search strategy is shown in Fig. 1 and the Prisma Checklist 2020 (*Supplementary material*). The characteristics of the included studies are summarized in Table 1. Among the 117 articles in Pubmed, Scopus, and CENTRAL that were retrieved, forty-four studies [5, 6, 13–54] were included in the qualitative and thirty-nine in the quantitative synthesis [5, 6, 13–36, 42–54]. The level of agreement between the two reviewers was “almost perfect” (kappa = 0.946; 95% CI:0.885, 1.000). The study design was randomized-controlled in eight studies [5, 6, 25, 37–39, 41, 49], and retrospective in thirty-six studies [13–24, 26–36, 40, 42–48, 51–54]. Propensity score matching (PSM) was performed in thirty-two studies [13–16, 18, 20–24, 26–28, 30–36, 42–48, 52–54]. The included studies were conducted in UK [13–15, 35, 43, 44], Italy [16, 19, 37], Australia [17], USA [21, 24–27, 30–34, 36, 38, 40, 47, 48], Canada [19, 20, 22, 28, 42, 45, 50, 53], Japan [51, 52, 54], Sweden [29], Portugal [46], Serbia [41], and three were multinational [5, 6, 49]. The studies were published between 1995 and 2022. The total sample size was 180,459 patients (MAG:56,175; SAG:124,284). The baseline characteristics and the NOS assessment of the included studies are provided in Table 1. The mean follow-up period ranged from 1 to 14 years. Figure 2a, b shows the qualitative assessment with the ROBINS-I tool. The authors’ main concerns are related to biases owing to the selection of participants and performance. The qualitative assessment of RCTs is demonstrated in *Table S1*.

**Table 1:** Baseline characteristics of the studies that were finally included in the meta-analysis

Study ID, year	Country	Study design	Patients, n MAG/SAG	Age, mean ± SD MAG/SAG	Female sex, n (%) MAG/SAG	EF, Mean ± SD, MAG/SAG	COPD, n (%) MAG/SAG	DM, n (%) MAG/SAG	Type of arterial grafts except for LITA, n (%)	OPCAB, n (%) MAG/SAG	Number of grafts, Mean ± SD MAG/SAG	Follow-up, Mean ± SD	NOS
Dewar 1995 [22]	Canada	R-PSM	377/765	60 (32–83)	Totally 185 (16)	NR	NR	NR	RITA:100%	NR	NR	7	8
Myers 2000 [38]	USA	RCT	81/80	63/63	18 (64)/ 20 (64)	NR	NR	11 (14)/12 (15)	RITA:100%	NR	NR	NR	–
Cohen 2001 [20]	Canada	R-PSM	478/ 956	61 ± 9/ 61 ± 9	76 (16)/152 (16)	NR	23 (5)/40 (4)	160 (34)/238 (25)	RA:100%	NR	3 ± 1/3 ± 1	36	7
Muneretto 2003 [37]	Italy	RCT	100/100	67 ± 9/68 ± 8	27 (27)/25 (25)	NR	19 (19)/22 (22)	41 (41)/40 (40)	RITA and RA	NR	3 ± 1/3 ± 1	1 ± 0.3	–
Hirottani 2003 [51]	Japan	R	179/124	65 ± 8/64 ± 9	42 (23)/31 (25)	48 ± 15/49 ± 16	NR	179 (100)/124 (100)	RITA:100%	NR	4 ± 1/3 ± 1	10	7
Calafiore 2004 [18]	Italy	R-PSM	570/570	61 ± 8/ 61 ± 9	110 (19)/100 (18)	59 ± 13/ 59 ± 14	16 (3)/17 (3)	138 (24)/138 (24)	RITA:100%	185 (33)/138 (24)	3 ± 1/3 ± 1	7 ± 5	8
Lytle 2004 [36]	USA	R-PSM	1152/ 1152	58 ± 8/ 58 ± 8	139 (12)/158 (14)	NR	NR	137 (12)/141 (12)	RITA:100%	NR	NR	NR	8
Lawton 2005 [32]	USA	R-PSM	294/294	62 ± 10/64 ± 10	294 (100)/294 (100)	48 ± 10/46 ± 12	NR	155 (53)/141 (48)	RA:100%	NR	NR	4 ± 2/4 ± 2	8
Guru 2006 [28]	Canada	R-PSM	5466/ 47,214	NR	875 (16)/9,915 (21)	NR	383 (7)/3,777 (8)	1,367 (25)/12,276 (26)	RITA and RA	NR	NR	5/6	8
Carrier 2009 [19]	Canada	R	1235 /5,420	61 ± 9/ 68 ± 8	199 (16)/ 1579 (29)	NR	NR	254 (21)/ 1696 (31)	RITA:100%	NR	NR	6	8
Nasso 2009 [39]	Italy	RCT	611 / 205	NR	346 (57)/120 (59)	NR	166 (27)/57 (28)	228 (37)/78 (38)	NR	NR	NR	3	–
Kurlansky 2010 [30]	USA	R-PSM	2,215/ 2,369	62.9 ± 10/68 ± 9	329 (15)/608 (26)	NR	NR	461 (21)/646 (27)	RITA:100%	NR	NR	13 (6–32)/11 (6–3)	8
Goldman 2011 [25]	USA	RCT	366/367	61 ± 8/62 ± 8	1 (1)/ 5 (1)	NR	NR	154 (42)/153 (42)	RA:100%	41 (11)/48 (13)	NR	5	–
Locker 2012 [34]	USA	R-PSM	1187/ 7435	58 ± 9/68 ± 9	179 (15)/1844 (25)	57 ± 11/55 ± 14	(7)/(12)	(18)/(34)	RITA and RA	(3)/(4)	NR	8 ± 5	7
Benedetto 2013 [13]	UK	R-PSM	936/8069	65 ± 10/68 ± 9	(20)/(18)	NR	(12)/(11)	(10)/(12)	RA:100%	(37.6)/(2.7)	3 ± 1/3 ± 1	6 ± 4	8
Lin 2013 [33]	USA	R-PSM	260/ 260	71 ± 9/ 71 ± 10	79 (30)/77 (30)	54 ± 14/ 53 ± 16	33 (13)/39 (15)	101 (39)/91 (34)	RA:100%	43 (17)/47 (18)	3 ± 1/3 ± 1	9 (6–12)	8
Parsa 2013 [40]	USA	R	728/16,881	59/64	144 (20)/ 4811 (29)	0.5/0.5	28 (4)/1,384 (8)	107 (15)/ 5,047 (30)	RITA:100%	NR	3/3	25	7
Benedetto 2014 [14]	UK	R-PSM	750/3445	NR	(81)/(11)	NR	58 (8)/365 (11)	119 (16)/1086 (32)	RITA:100%	538 (72)/2235 (65)	NR	5 ± 3	8
Buxton 2014 [17]	Australia	R	2988/ 786	65 ± 10/71 ± 8	572 (19)/193 (25)	NR	114 (4)/26 (3)	859 (29)/321 (41)	RITA and RA	NR	3 ± 1/4 ± 1	NR	7
Garatti 2014 [23]	Italy	R-PSM	209/243	48 ± 8/50 ± 7	10 (5)/10 (4)	52 ± 9/ 52 ± 10	8 (4)/7 (3)	31 (15)/34 (14)	RITA and RA	NR	3 ± 1/4 ± 1	14 ± 3/14 ± 4	8
Mohammadi 2014 [53]	Canada	R-PSM	111/111	56 ± 9/57 ± 10	12 (11)/ 14 (13)	34 ± 5/ 32 ± 7	17 (15)/18 (16)	17 (16)/18 (16)	RITA:100%	NR	NR	20	8
Pullan 2014 [43]	UK	R-PSM	2,940/ 7006	62 ± 62/65 ± 65	NR	NR	NR	1147 ± 39/2382 ± 34	RA:100%	1,999 ± 68 /1261 ± 18	3 ± 3/4 ± 3	7 ± 7/ 7 ± 7	8
Schwann 2014 [47]	USA	R-PSM	2,794/2,794	Similar	Similar	Similar	Similar	Similar	RA:100%	NR	Similar	NR	8
Grau 2015 [27]	USA	R-PSM	1,544/5,122	NR	NR	NR	NR	NR	RITA:100%	NR	4 ± 1/4 ± 1	15	8
Kinoshita 2015 [52]	Japan	R-PSM	412/412	72 ± 8/72 ± 8	80 (19)/90 (22)	NR	82 (20)/91 (22)	245 (59)/233 (57)	RITA:100%	100%	4 ± 1/3 ± 1	12	8
LaPar 2015 [31]	USA	R-PSM	1333/1,333	56 ± 10/ 59 ± 10	191 (14)/249 (19)	0.6 [0.50–0.60]/ 0.6 [0.50–0.60]	NR	243 (18)/465 (35)	RITA:100%	NR	3 ± 3/3 ± 3	NR	7
Petrovic 2015 [41]	Serbia	RCT	100/100	56 ± 6/57 ± 7	27 (27)/ 27 (27)	49 ± 11/48 ± 11	9 (9)/8 (8)	39 (39)/43 (43)	RA:100%	NR	3 ± 1/ 3 ± 1	8	–
Raja 2015 [44]	UK	R-PSM	RA:779 /RITA: 747	RA:62 RITA: 60	RA:125 (16) RITA: 82 (11)	NR	RA:55 (7) RITA: 60 (8)	RA:242 (31) RITA: 120 (16)	RA:51% RITA:49%	NR	NR	8	8
Schwann 2016 [48]	USA	R-PSM	3736/4,484	Similar	Similar	Similar	Similar	Similar	RA:82.8% RITA:17.2%	NR	Similar	0–16	8
Yamaguchi 2016 [54]	Japan	R-PSM	1309/1309	Similar	Similar	Similar	Similar	Similar	RIMA:809 RA:224	Similar	Similar	12	8
Benedetto 2017 [15]	UK	R-PSM	3,026/9606	NR	327 (11)/1773 (19)	NR	127 (4)/769 (8)	405 (13)/1773 (19)	RA:2,001 (66) RITA:755 (25) RITA + RA: 270 (9)	1,818 (60)/4412 (46)	NR	8 ± 5	8
Bisleri 2017 [16]	Italy	R-PSM	315/201	77 ± 6/79 ± 7	89 (28)/61 (30)	NR	58 (18)/24 (12)	155 (49)/96 (48)	RITA: 131 (75) RA: NR	62 (37)/36 (21)	2.5 ± 0.5/2.6 ± 0.4	7 (0–10)	8
Pu 2017 [42]	Canada	R-PSM	5,580/14,496	60 ± 9/ 68 ± 9	586 (11)/ 2803 (19)	NR	816 (15)/3028 (21)	1,650 (30)/5771 (40)	RITA and RA	NR	NR	4/4	8
DeSimone 2018 [21]	USA	R	1482/ 46,502	NR	276 (19)/12,044 (26)	NR	122 (8)/5,441 (12)	239 (16)/15,857 (34)	RITA:100%	142 (10)/4,464 (10)	NR	NR	7
Goldstone 2018 [26]	USA	R-PSM	5,866/ 53,566	62 ± 11/67 ± 10	863 (15)/13,636 (26)	56 ± 12/52 ± 14	NR	2,077 (35)/24,481 (46)	RITA:1,570 (27%) RA:4,272 (73%)	NR	NR	5.3 (4–7)	8
Janiec 2018 [29]	Sweden	R	1,898/ 46,343	RA: 65 ± 10 RIMA: 64 ± 9/ 66 ± 8	RA:277 (27) RIMA: 146 (17)/8879 (19)	NR	98 (5)/2551 (7)	418 (22)/ 11,077 (24)	RITA:862 (45) RA:1,036 (55)	132 (7)/ 1,103 (2)	NR	RA:11 ± 4 RITA: 6 ± 5/ 9 ± 4	8
Luthra 2018 [35]	UK	R-PSM	1238/ 2757	66 ± 10/66 ± 10	219 (18)/505 (18)	NR	152 (12)/362 (13)	342 (28)/724 (26)	RITA and RA	NR	4 ± 1/4 ± 1	6/5	8
Rocha 2018 [45]	Canada	R-PSM	8,629/ 8,629	NR	NR	NR	NR	NR	RITA and RA	NR	NR	4	7
Saraiva 2018 [46]	Portugal	R-PSM	936/1488	59 ± 10/67 ± 9	124 (13)/336 (23)	NR	52 (6)/93 (6)	339 (36)/675 (45)	RITA:100%	608 ± 65/381 ± 26	NR	5	8
Taggart 2019 [5]*	Multinational	RCT	1548/1,554	64 ± 9/ 64 ± 9	230 (15)/216 (14)	NR	NR	371 (24)/363 (23)	RITA:100%	NR	NR	10	–
Tam 2020 [50]	Canada	R-PSM	2,961/7954	66 ± 10/69 ± 9	2,961 (100)/7,954 (100)	NR	237 (8)/865 (11)	1373 (46)/3812 (48)	RITA and RA	1190 (40)/ 893 (11)	3 ± 1/3 ± 1	5 (3–8)	8
Gaudino 2021 [24]	USA	R-PSM	12,629/50,773	61 ± 10/66 ± 10	2101 (14)/13 146 (86)	NR	412 (20)/ 3451 (26)	898 (43)/ 6491 (49)	RITA and RA	465 (22)/3062 (23)	NR	6 (4–9)	8
Taggart 2022 [49]*	Multinational	RCT-Post-hoc	405/311	64 (58–69)/64 (57–70)	74 (18)/62 (20)	NR	30 (7)/17 (6)	405 (100)/311 (100)	RITA:100%	NR	NR	10	
Thuijs 2022 [6]	Multinational	RCT– Post-hoc	465/ 1001	62 ± 10/67 ± 9	64 (14)/224 (22)	NR	34 (7)/89 (9)	141 (31)/361 (36)	RITA:73% RA:41%	101 (22)/146 (15)	3 ± 1/3 ± 1	13	–

The Newcastle–Ottawa Scale (NOS) was used for assessing the quality of non-randomized studies. The highest quality studies are awarded up to 9 stars

*R* retrospective, *PSM* propensity score matching, *RCT* randomized-controlled trial, *NR* not reported, *MAG* multiple arterial grafting, *SAG* single arterial grafting, *OPCAB* off-pump coronary artery bypass, *RITA* right internal thoracic artery, *RA* radial artery, *SD* standard deviation

^*^Data presented are on the same population but were incorporated only for subgroup analyses. No incorporation of double population has been made

### Primary endpoints: OS and event-free OS

**Fig. 3:**
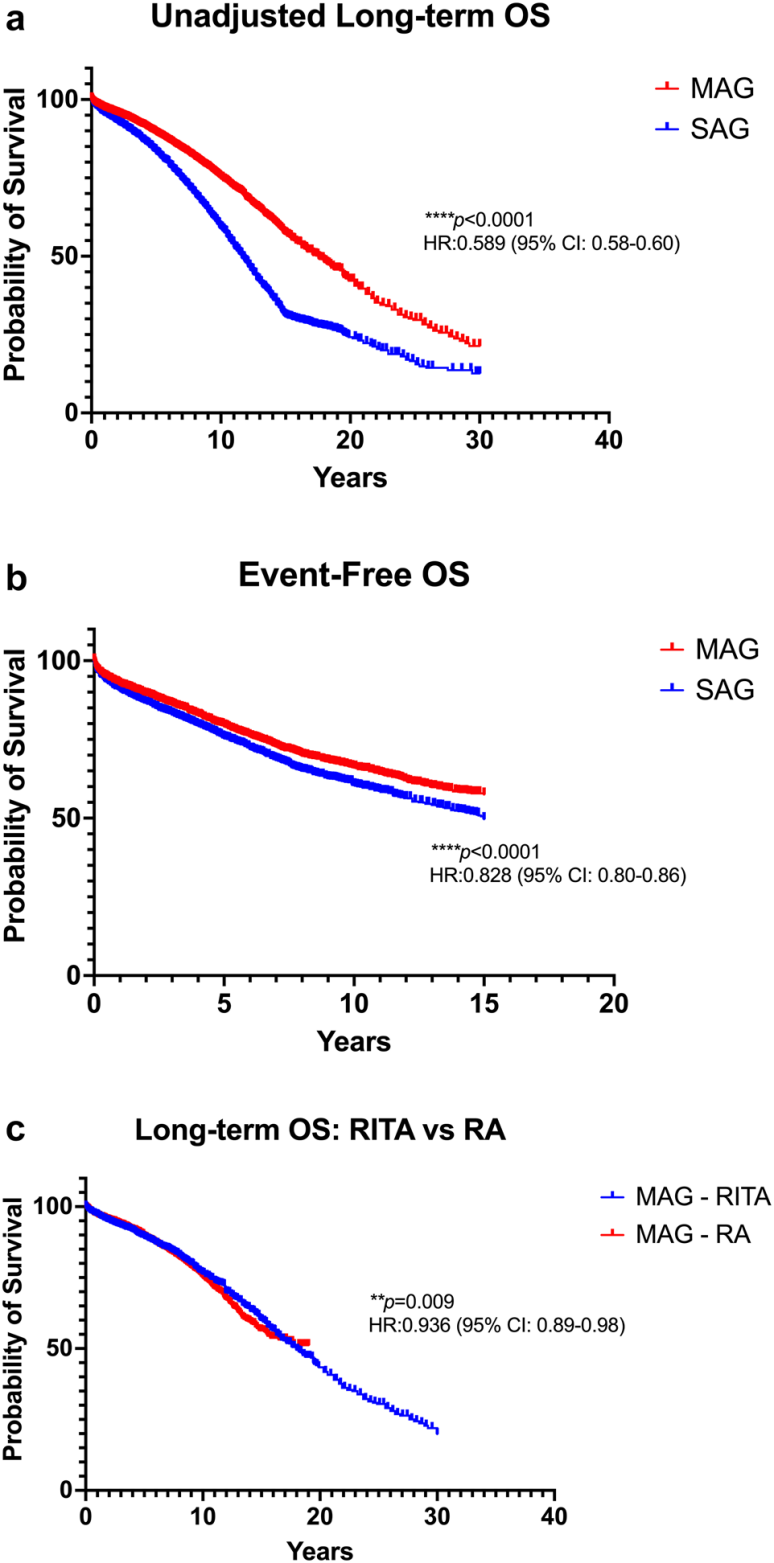
Pooled Kaplan–Meier curves comparing multiple arterial grafting (MAG) versus single arterial grafting (SAG) for **a**. overall survival (OS), **b**. event-free OS, and **c**. difference in OS between right internal thoracic artery (RITA) and radial artery (RA) as a second arterial conduit. *CI* confidence interval; *HR* hazard ratio

Figure 3A depicts the pooled Kaplan–Meier curves for overall survival in the total, unadjusted for risk factors, population. Patients in the MAG group demonstrated a significantly higher OS (HR:0.59; 95% CI:0.58–0.60; *p* < 0.0001). Median OS was 17.54 years for the MAG group and 11.63 years for the SAG group. Figure 3b depicts the pooled Kaplan–Meier curves for event-free survival, incorporating data from 47 to 376 patients (MAG:23, 569 patients; SAG:23,807 patients). Patients in the MAG group were associated with a significantly higher event-free OS (0.83; 0.80–0.86; *p* < 0.0001).

### Secondary endpoints: RIMA vs RA as a second arterial conduit in terms of OS

Figure 3C depicts the pooled Kaplan–Meier curves regarding OS for patients that received either a RITA or a RA as a second arterial conduit. The data of 31,178 patients (RITA:13,575 patients; RA:20,245 patients) were incorporated. Patients in the RITA group demonstrated a significantly higher OS (HR:0.936; CI:0.89–0.98; *p* = 0.009).

### Subgroup analyses

**Fig. 4:**
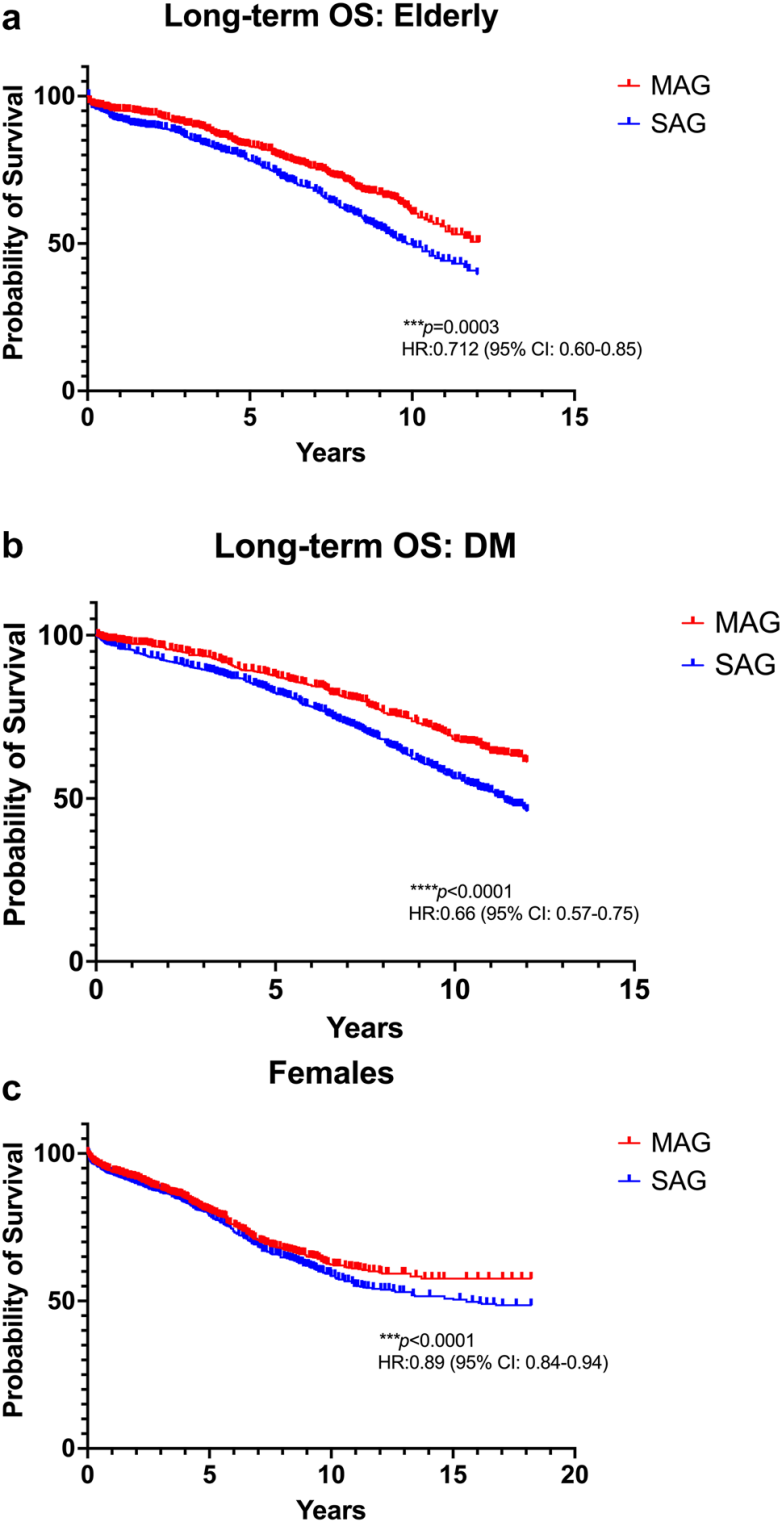
Subgroup pooled Kaplan–Meier curves regarding overall survival (OS) for patients **a**. > 70 years, **b**. with diabetes mellitus (DM), and **c**. female. *CI* confidence interval, *HR* hazard ratio

Finally, we performed subgroup analyses comparing MAG vs SAG in (a) patients > 70 years, (b) patients with DM, and (c) female patients. MAG was superior in terms of OS in all three subgroup analyses, as demonstrated in Figs. 4a, b, c, and Table 2.

**Table 2:** Summary of the primary and secondary endpoints, along with the sensitivity and subgroup analyses

Endpoint	*N*	HR (Mantel–Haenszel)	95% CI	*p *value
Unadjusted OS	180,459	0.589	0.58–0.60	< 0,0001
Adjusted OS	80,036	0.706	0.69–0.73	< 0,0001
Event-free OS	47,376	0.828	0.80–0.86	< 0,0001
OS RITA vs RA	31,178	0.936	0.89–0.98	0,009
OS Age > 70 years	1452	0.712	0.60–0.85	0.0003
OS Diabetes mellitus	2516	0.656	0.57–0.75	< 0,0001
OS Females	14,834	0.889	0.84–0.94	< 0.0001

*OS* overall survival, *RITA* right internal thoracic artery, *RA* radial artery, *HR*  hazard ratio;95% *CI* 95% confidence intervals; *n* = numbers

### Sensitivity analysis

**Fig. 5:**
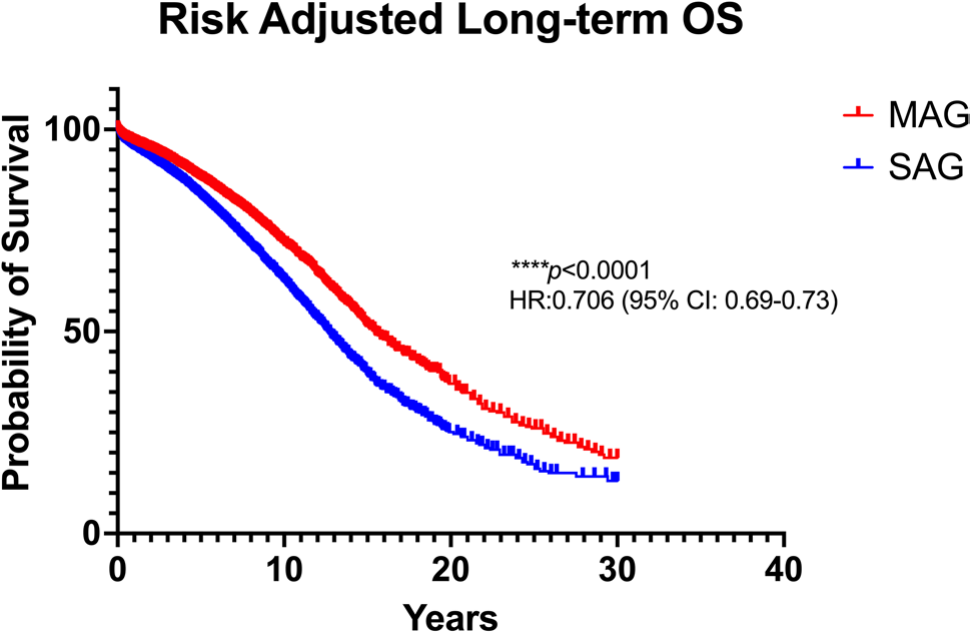
Risk-adjusted pooled Kaplan–Meier curve regarding overall survival (OS). *CI* confidence interval, *HR* hazard ratio

No difference in the survival outcomes was found after performing the leave-one-out sensitivity analysis. This should be especially highlighted in the secondary outcomes, given the longer follow-up of the RITA group. Nonetheless, it should be noted that in the subgroup analysis regarding females, there was a significant superiority of MAG over SAG when excluding either the study by Pullan et al. [43] or Gaudino et al. [24], which is in accordance with previous evidence [54]. In fact, the outcomes did not change, after curing data to include comparable follow-up periods. In a second step, we adjusted the patient data for potential cofounders (age, gender, comorbidities, prior myocardial infraction (MI), prior cardiac intervention/surgery, ejection fraction, presence/absence of LMCAD) regarding median OS. The outcomes were similar to the total analysis, as demonstrated in Fig. 5.

## Discussion

The present meta-analysis identified forty-three articles and provides additional value to the existing literature since it is the first meta-analysis pooling reconstructed time-to-event data of 180,459 patients at the independent patient level and producing pooled Kaplan–Meier curves. According to our outcomes, MAG is associated with enhanced long-term survival outcomes compared to SAG. These results are further validated by the sensitivity/subgroup analyses. Although a previous meta-analysis [3] was conducted in 2019 (study period until 12/2018), it was associated with several methodological limitations, such as the absence of important subgroup analyses (gender, DM), while no Kaplan–Meier curves were constructed, and the data extraction was not at the patient level.

Defining the optimal CABG grafting strategy is crucial to enhance quality in terms of clinical outcomes, along with economic efficiency. Nonetheless, the recent evidence on long-term survival provided by large RCTs has been contradictory [25, 49] and the conundrum still exists. In the same context, outcomes from the ROMA trial are expected no earlier than 2025 [7]. According to the present study, patients in the MAG group demonstrated higher OS in both the unadjusted and adjusted analyses. Furthermore, they were associated with superior event-free OS compared with those patients receiving SAG.

The evidence provided in the literature regarding the comparison between the RITA and RA as a second arterial conduit is discordant. Two previous meta-analyses performed by the same team [55, 56] demonstrated contradicting outcomes. According to the earlier one [55], RITA was associated with a 25% relative reduction in the risk of long-term mortality. On the other hand, the most recent meta-analysis showed similar long-term survival between the two arterial conduits [56]. According to our outcomes, which were produced by building a pooled Kaplan–Meier curve, RITA was associated with a higher long-term survival when used as a second arterial conduit compared with RA. Potential reasons underlying these discrepancies might be (a) the different sample size and follow-up period (the present study is the biggest incorporating 180,459 patients), (b) the different data extraction (reconstructed time-to-event data in the present study) and statistical methods (construction of pooled Kaplan–Meier curves), (c) differences regarding the surgical technique (e.g., skeletonized or not regarding RITA, differences in RA harvesting protocol), (d) potential differences in treatment protocol regarding the extend of stenosis of target vessels for RA conduits, (e) differences in the post-discharge treatment protocols, and (f) potential differences regarding baseline characteristics of the included patients in spite of the risk-adjusted nature of the comparisons. In fact, a crucial point when using the RA for CABG is the degree of target vessel stenosis. It has been shown that the patency rate of RA grafts is strongly influenced by the degree of target stenosis [57].

The present meta-analysis also demonstrated the superiority of MAG over SAG for two groups of high-risk patients, those aged > 70 years and those with DM. Evidence remains contradicted regarding the usefulness of MAG in elderly patients, along with the cutoff age to define a patient as elderly [58]. In the present meta-analysis, we used the age of 70 years as a cutoff point and we demonstrated that MAG is superior to the SAG strategy in terms of OS in the > 70 years group. This outcome is in accordance with previous evidence [59]. However, it would be interesting to examine the value of MAG in elderly patients with reduced EF, an endpoint that was out of the scope of the present meta-analysis. In the same context, Chikwe et al. [60] analyzed the New Jersey registry of 26,000 patients and found no significant benefits of MAG in patients > 70 years with reduced EF. The potential value of MAG in patients with DM has been another debatable topic. In fact, according to a recent post hoc analysis of the ART trial [49], MAG is associated with higher OS in patients with DM, independently of the type of DM, which is in accordance with our findings.

Another subgroup analysis we performed was the difference between the two strategies in terms of OS in female patients. Generally, women represent an under-represented patient group in observational and randomized CABG studies. For instance, women represented a 15% ratio of the 3,102 patients incorporated in the ART study [49]. Nonetheless, women have significant differences in biology and baseline characteristics compared with male patients. According to our outcomes, MAG was again superior to SAG in terms of OS in female patients. Nonetheless, this outcome was sensitive to the small-study effect, demonstrating when we performed the leave-one-out sensitivity analysis. These results are in agreement with the outcomes of a previous meta-analysis by Robinson et al. [61].

The limitations of the current meta-analysis reflect the limitations of the studies included. Although the majority of the studies were retrospective in nature, they provided either risk-adjusted or PSM analyses. Furthermore, eight studies were RCTs. In addition, the included studies are related to biases related to the selection of participants and performance. Moreover, the differences among institutions regarding the selection criteria, treatment protocols, and perioperative management pose certain limitations. In fact, the selection criteria were not homogenous and may have been based on the patients’ clinical attributes and status, thus posing a selection bias that could not be adjusted in the present study. Finally, patient data were gathered from Kaplan–Meier-derived data, thus limiting our ability to perform further multivariable analyses.

On the other hand, the strengths of this study include (a) the clear data extraction protocol, (b) the well-specified inclusion–exclusion criteria, (c) the search that was performed in three different databases, (d) the quality assessment of the included studies, (e) the detailed presentation of the results of data extraction and analysis, (f) the extraction of survival data at the level of the independent patient, and (g) the performance of sensitivity and subgroup analyses.

## Conclusion

In the context of patients with central CAD undergoing isolated CABG, MAG is superior to SAG in terms of median OS and event-free OS. Furthermore, MAG was also superior for patients > 70 years, females, or patients with DM. Finally, RITA was superior to RA as a second arterial conduit on long-term OS. The present evidence represents the best currently available level of evidence and should be used as a bridge until the publication of ROMA trial outcomes.

## Supplementary Information

Below is the link to the electronic supplementary material.

Supplementary file1 (DOCX 28 KB)

## Data Availability

The data supporting this study are available from the corresponding author, upon reasonable request.
