# Supplementary material for: Differences in long-term survival outcomes after coronary artery bypass grafting using single vs multiple arterial grafts: a meta-analysis with reconstructed time-to-event data and subgroup analyses
Source: Gen Thorac Cardiovasc Surg. 2022 Nov 17;71(2):77–89. doi: 10.1007/s11748-022-01891-7 (PMC9886578; doi:10.1007/s11748-022-01891-7)
Supplement: Supplementary file 1 — Supplementary file1 (DOCX 28 KB) [file 11748_2022_1891_MOESM1_ESM.docx]

| **References** | **Random sequence generation** | **Allocation concealment** | **Blinding of participants and personnel** | **Blinding of outcome assessment** | **Incomplete outcome data** | **Selective reporting** |
| --- | --- | --- | --- | --- | --- | --- |
| Goldman 2011 [25] | Low risk | High risk | Low risk | Low risk | Low risk | High risk |
| Muneretto 2003 [37] | Low risk | Unknown | Low risk | Low risk | High risk | Unknown |
| Myers 2000 [38] | Low risk | High risk | Low risk | Low risk | Low risk | Low risk |
| Nasso 2009 [39] | Low risk | Unknown | Low risk | Low risk | Low risk | Low risk |
| Petrovic 2015 [41] | Low risk | Unknown | Low risk | Low risk | Low risk | Low risk |
| Taggart 2019 [5] | Low risk | High risk | Low risk | Low risk | Low risk | Low risk |
| Taggart 2022 [49] | Low risk | High risk | Low risk | Low risk | Low risk | Low risk |
| Thuijs 2022 [6] | Low risk | Low risk | Low risk | Low risk | Low risk | Low risk |

The RCTs were assessed for their methodological quality with the tools that are used to evaluate the risk of bias according to the Cochrane Handbook for Systematic Reviews of Interventions
